# Study protocol—an exploratory trial on health promoting schools at Dutch secondary schools

**DOI:** 10.5334/ijic.821

**Published:** 2012-07-24

**Authors:** Vincent Busch, Johannes Rob Josephus De Leeuw, Augustinus Jacobus Petrus Schrijvers

**Affiliations:** Julius Center for Health Sciences and Primary Care, University Medical Center Utrecht, The Netherlands; Julius Center for Health Sciences and Primary Care, University Medical Center Utrecht, Room Str. 5.107, P.O. Box 85500, HP Str 6.131, Universiteitsweg 100, 3508 GA, Utrecht, The Netherlands; Julius Center for Health Sciences and Primary Care, University Medical Center Utrecht, Room Str. 5.123, P.O. Box 85500, HP Str 6.131, Universiteitsweg 100, 3508 GA, Utrecht, The Netherlands

**Keywords:** health education, youth health, protocol

## Abstract

**Background:**

Recent studies show adolescent health-related behaviours to co-occur synergistically. This paper describes the study design for an exploratory trial on the effects of a comprehensive, whole-school health promoting school intervention. This intervention tackles seven different behavioural domains simultaneously via a combination of education, creating a healthy environment and introducing healthy behavioural policies. Additionally, extensive partnerships are formed between schools, parents, neighbourhoods and youth health authorities to coordinate health promotion efforts.

**Study design and data collection methods:**

The intervention will be implemented at two secondary schools. Results will be compared with two control schools (n≈1500). The intervention’s effectiveness in changing student behaviours as well as physical and psychosocial health status along with qualitative lessons learned on the integration of youth health care services and school health education practices are the main aimed outcomes of this study. Data are collected via a mixed methods design combining an annual youth health (behaviour) monitor with a qualitative process evaluation via interviews with key stakeholders.

**Data analysis:**

A multilevel analysis is performed combined with a systematic analysis of qualitative interview data.

**Conclusions:**

This study will produce an evaluation of a comprehensive health promoting school intervention that combines an integrated approach of schools, neighbourhoods, families and youth health services to improve adolescent health.

## Introduction

Unhealthy behaviours such as alcohol use, smoking, unhealthy nutrition or excessive time spent behind screens (e.g. the television, computer or game console) show alarming trends in The Netherlands and the rest of the developed world [1, 2]. Children and adolescents are a particularly vulnerable subpopulation in this respect, with half of all adolescents being involved in *at least* one or more unhealthy behaviours [3]. Many of these unhealthy behaviours are known to originate in adolescence and consequently pose a gateway to poor adult health [4]. Moreover, recent literature suggests that these behaviours act as being associated with each other instead of acting independently. In several recent studies this clustering is hypothesized to act on one’s health interactively, i.e. individual behaviours yield greater effects when present together than their individual sums would be expected to [1, 5–8].

Therefore, research increasingly suggests that multi-behavioural targeting interventions hold the most promising future perspectives when it comes to influencing unhealthy behaviours via preventive action [7–10]. It is suggested that these interventions hold the advantage of also simultaneously influencing behaviours outside primarily targeted ones; for example, intervening in adolescents’ smoking behaviour, while then simultaneously also affecting their alcohol consuming behaviour. Several recent studies have shown promising examples of this principle and they show interesting developments for their practical implications [7, 8, 11]. However, unfortunately, most empirical intervention studies still focus on targeting one single risk factor or behaviour at a time, which often causes failure to take into account much of the real world relevance that is crucial to gaining valuable insights into evaluating such interventions in a real-life educational setting [9, 12–15]. They do not sufficiently assess the intervention’s effects in a comprehensive fashion. Therefore, several recent papers of different types of studies, such as those by James Prochaska [10], Judith Prochaska et al. [13, 16–19] and Alfredo Morabia and Costanza [20, 21], have recently pressed the importance of persisting to study health (interventions) in real world settings to achieve the needed *meaningful* progress in (school) health promotion research. Also among them are several (trial) studies by Brian Flay [22–24], who states that: “*problem/risky behaviours, unhealthy behaviours, anti-social behaviour, poor mental health, and poor academic achievement should be addressed by a comprehensive, coherent, and integrated approach, rather that the disjointed approach to prevention and promotion taken by education today*” [22, p. 4]*.*

### Pilot study

**Figure 1:**
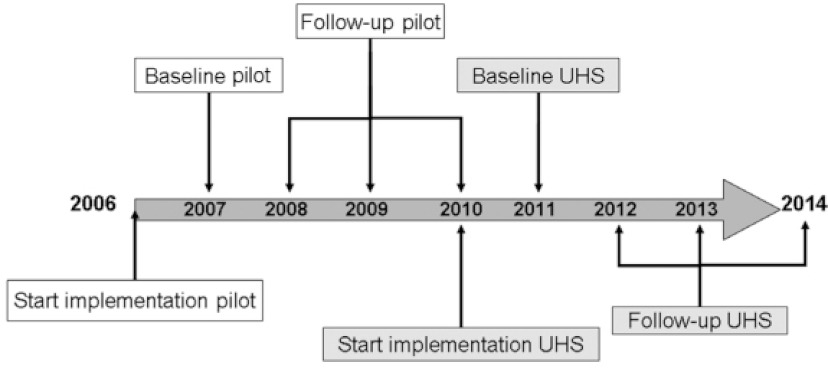
Utecht Healthy School timeline.

In 2006 a health promoting school pilot was developed and implemented on a secondary school in The Netherlands (see Figure 1). It targeted young adolescents of the first three grades of a Dutch secondary school (approx. 11–16 year olds). The desire to pursue a more comprehensive, evidence-based health promoting school concept drove the development of this pilot; a desire that originated due to persistent complaints from parental and teachers’ about the students’ unhealthy lifestyle. These complaints were a result of persisting problems with, among other things, students’ (extreme) alcohol use and bullying. Therefore, this particular group of young adolescents was chosen as target population for this pilot, although the authors realize that much progressive research has been performed on intervening in younger children by pioneers in the field, such as Lawrence St. Leger [25].

The pilot was inspired by the *Schools for Health in Europe*’s (SHE)* Whole School Approach (WSA)* [26–28]. This model promotes a comprehensive *total-life-approach* instead of focusing on a single dimension of child health, such as school life or family life. Following the WSA’s framework, the pilot consisted of several components [29]. First, several evidence-based curricular components were *integrated* into the pre-existing curriculum, instead of simply replacing or adding to it. These curricular changes brought about that: 1) health education constituted a significantly larger portion of the school’s curriculum targeting several topics more in-depth than is common in Dutch secondary schools, 2) health education topics were structurally embedded into the curriculum, instead of being disjointed, sporadic projects, 3) certain topics were targeted in a clustered fashion, such as substance use behaviours or screen time behaviours, 4) evidence-based methods were used in health education, which is also uncommon practice in Dutch secondary schools, 5) a strong focus was placed on students’ personal skill development, refusal skills training, peer education and genuine, active participation [30], and 6) annual monitoring of student health behaviours and health status took place via a self-report questionnaire to fine-tune school policy and create annual spear points.

In total, seven health related behaviours were simultaneously targeted in the pilot, selected based on academic literature, namely nutrition, physical exercise, substance use (alcohol and marijuana), smoking, bullying, sexual behaviour and screen time behaviours [watching television, pc/internet use and (online) gaming]. The most notable ‘newcomers’ in this group of behaviours are the *screen time behaviours*. They were chosen due to their addictive potential and their importance to both physical and psychosocial adolescent health, as stated in recent studies [31].

In addition to the curriculum changes, different (health) behavioural school policies (e.g. no smoking policy) were implemented, together with the creation of a healthy, supportive school environment (e.g. no candy machines at school, a healthy school canteen). Also, extensive partnerships with the students’ social environment (with parents, the local police, local supermarkets, etc.) were formed to facilitate the development of a healthy environment for the students outside of school. In addition, the municipal health services were re-oriented towards school and prevention by e.g. creating a faster referral service to local primary health care services. Therefore, the municipal health services form close partnerships with the school to be involved with the format and content of the pilot in order to fine-tune it to recent evidence-based or best practices.

### Implementation of the pilot on the school

Instead of standardizing the pilot, the school adopted a tailored, school-specific implementation approach, based on theory and literature. To achieve this, the school first prioritized what health-related behaviours and topics a health promoting school should focus on by applying a baseline student-questionnaire/monitor to map all relevant behaviours and issues. Afterwards, these outcomes were discussed with the parents, via the parent council, in order to place emphasis on appropriate topics and to initialize the process of installing a health promoting school steering group to guide the prioritizing and further implementation. This steering group consisted of a representative of the student council, the parent council, of the teaching staff, of the school board, of the school policy makers and several researchers and policy-makers from Dutch knowledge/academic institutes and the regional public health authorities. The ‘academic’ participants were involved to ensure the evidence-based nature of the incorporated materials/intervention parts, while the ‘school’ participants were responsible for the tailoring of these materials/intervention parts to fit the specific school context.

### First results of the pilot study

Recently, several papers have been published about the pilot study and student behaviours on the school [11, 32]. First, De Leeuw and colleagues reported on the (online) gaming and internet/pc use behaviours [11]. In addition, Sterkenburg and colleagues recently described the relationship between bullying, psychosocial health and happiness at the school [32]. Furthermore, other preliminary results suggest positive effects with regard to smoking, alcohol use, binge drinking and cannabis use.

### Lessons learned during the pilot study

The pilot study’s most notable lessons learned mainly regarded the novel multi-actor nature of the intervention, involving school, parents, neighbourhood and municipal health services. It was shown to be important to 1) assess the feasibility of close cooperation with the students’ parents, 2) to facilitate a close cooperation with the municipal/regional health services in order to keep the educational modules and methods evidence-based and up to date, 3) fine-tune initiatives within the school’s neighbourhood (e.g. surrounding supermarkets), and 4) to provide the teaching staff with in-service trainings, in order to provide them with the necessary tools and competencies to optimally function in this ‘new’ curriculum and context. During the pilot study, the municipal health services showed great willingness to provide schools with such help e.g. by means of delivering evidence-based educational materials and teacher in-service trainings. All these partnerships facilitated an integral, more intensified approach to local health promotion, through integration of school, local environments and primary health care services.

After viewing the positive effects of the pilot on the school, the current, follow-up study was initiated to determine the feasibility of the intervention at more regular secondary schools with more ‘standard’ student demographics and school conditions. This study protocol presents the rationale and methodology for the exploratory trial study [33] of the pilot, named and from hereon referred to in relation to the follow-up study as the *Utrecht Healthy School (UHS)*.

## Methods

### Study design: an exploratory trial

The UHS remains similar in terms of format, content and implementation as the pilot, only targeting different schools in a different study setting. It will be implemented on two Dutch secondary schools, with two comparable schools serving as controls (n≈1500 students) in an exploratory trial study design [33].

**Figure 2:**
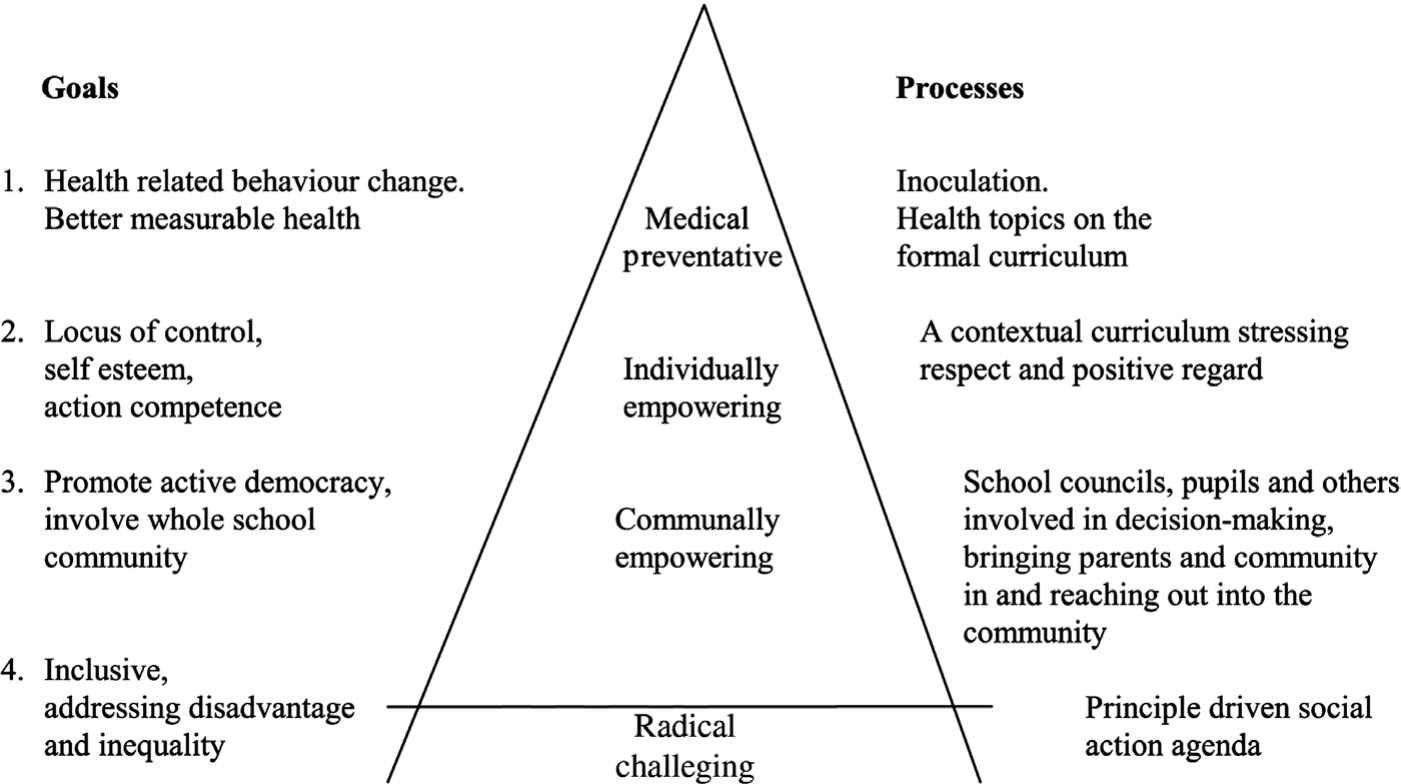
Health Promoting School hierarchy of goals and processes [35].

The main outcomes of the study will be the (health) behavioural change effects in students. In addition, the qualitative process evaluation components serve to optimally interpret the quantitative student behavioural change data in their proper context and to evaluate the UHS’ feasibility for implementation on relatively ‘standard’ schools in The Netherlands, selected as a convenience sample. Afterwards, comparable control schools were selected and incorporated in the study. Campbell et al. underscore the value of such a mixed methods approach, due to the complexity of the UHS [34]. Their model of “*continuum of evidence for complex interventions*” applies well to the current study, which resides in its Second Phase. In Phase II, the UHS study resides between a randomized controlled trial phase (Phase III) and the pilot phase (Phase I) [33]. During this phase the UHS’ effects and implementation processes are evaluated under more regular circumstances in an exploratory fashion via a small-scale, controlled intervention-control study. The main issues to take into account in such studies, according to Campbell et al. [33], are 1) ensuring a sound theoretical understanding of the problem, 2) realizing that a lack of intervention effect may reflect implementation failure rather than a genuine lack of program effectiveness, 3) the variability in individual level outcomes may reflect upon higher processes, 4) a single primary outcome may not make the best use of the data, which means that e.g. quantitative data can better be interpreted when accompanied by related qualitative data that may provide the necessary context, and 5) realizing that ensuring strict standardization may be inappropriate in this context. The UHS may work better when a certain degree of adaptation to local settings is permitted. As the model by Parsons and Stears illustrates (Figure 2), such processes are suggested to accompany the according goals of Health Promoting School interventions. During the implementation at the two intervention schools, an entire year has been spent to fine-tuning these processes. Flay also provides similar recommendations, specifically for these kinds of complex interventions, naming this phase/type of study a *prototype evaluation study* [36].

### Sample and procedures

Students from grade one to three (11–16 year olds) are questioned via an annual online questionnaire, completed individually, in-class. Survey procedures are designed to allow students to participate *voluntarily* and *confidentially*; prior to the survey students are extensively informed of this. These procedures were similar in the pilot project.

#### Student survey

The survey assesses a range of health behaviours, health outcomes and socio-demographic characteristics. Most items were derived from the validated Dutch conversion of the HBSC-survey [37, 38] (see Table 1). Non-HBSC items are the Compulsive Internet Use Scale [45] and the Videogame Addiction Test [40].

### Process evaluation

Periodic, semi-structured interviews are performed with the Healthy School Coordinator to gain insight into the (progress of the) implementation process. S/he is a central coordinating figure in each participating school. S/he forms the link between school-related health promotion activities, parents, neighbourhoods and the municipal health services. S/he assures that the SHE’s *whole-school approach* [26, 28] is followed. This level of cooperation between the education and primary health care sector on such an individual school level is uncommon in The Netherlands. Similar interviews are performed at the control schools with the central contact person to account for any changes in related curricular activities to allow for better data-interpretation. The process evaluation is thematically analyzed by means of the qualitative data analysis program NVIVO [44].

## Data analysis

### Main outcomes: student behavioural changes

Multilevel regression analyses and ANCOVA analyses will be performed to analyze changes in student health behaviours and health outcomes. The multilevel analyses will contain two levels: the school level and the individual student level.

#### Power calculations

Prior to this exploratory trial, the means and standard deviations of the main behavioural outcomes were estimated in the pilot study, based on previous Dutch studies to perform a power analysis for determining the study’s required sample size. As stated in literature, a ‘generic’ target of 10–15% points of detectable change suits a program such as this UHS-exploratory trial [46]. As stated by Babyak, for multiple regression analyses 15 persons/observations per parameter are to be included in the measurements for conservative power calculations [47]. With an expected response rate of >90% and 30 included parameters to be measured, a sample size of approximately 500 observations is required for a power of 0.80 at an alpha-level of 0.05 for statistical significance, whilst approximately 1500 students are expected to participate in the study. The sample size thus exceeds the needed requirements, but was chosen to guarantee the study results being sufficiently representative and to allow for sub-group analyses.

## Discussion

Recently several studies advocated the use of a *total life approach,* as promoted by SHE’s whole school approach. This approach aims to reach out beyond the borders of an adolescent’s ‘school life’ by incorporating the social environment(s), parents and the community [25, 27, 28]. Another aspect of this approach that is included in this approach is to make use of comprehensive interventions (including the creation of a healthy school environment and accordingly re-shaping behavioural policies) in contrast to ‘merely’ adding a standardized portion of health education to a given school curriculum/setting. The inclusion of a Healthy School Coordinator ensured that all whole school approach aspects were fine-tuned to and integrated within the school as well as with external parties. The most important aspect of this coordinating function is to steer the implementation and to guide the ‘tailoring process’ instead of implementing a highly standardized UHS. Both in the pilot study as well as in the current study the prioritization of the main *behaviours to tackle* and *outcomes to pursue* should be tackled by means of a questionnaire before and yearly after the start of the study. A recent successful large-scale example of such an approach was provided by Patton and all in their Gatehouse Project [48].

**Figure 3:**
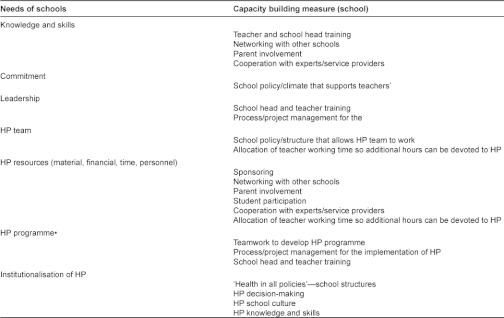
A summary of Guggleberger and Dür’s needs of schools and the capacity building measures for schools for Health Promoting School implementation [50].

To stimulate the sustainability of the UHS, the schools did not receive external financial or organizational support and kept their complete autonomy and own internal structures. Taking into account the lessons learned from Flay [36], Campbell et al. [33], Pawson et al. [49], Campbell et al. [34] and Parsons and Stears [35] the first full year of the intervention was spent on internal organization and integrating the intervention *into* the pre-existing school practices and curriculum. A summary of the capacities that are needed for successful implementation of such a comprehensive health promoting school program as the UHS, a summary of Guggleberger and Dür’s recommendations on this account is presented below (Figure 3).

The aspect that creates the intervention’s potential success (its comprehensiveness) also creates potentially difficulties in analyzing its effectiveness. The variety of factors that make up the equation of the student’s health in the intervention schools makes it challenging to clearly distinguish between successful and unsuccessful characteristics of the program. The authors have tried to signal possible confounding effects by a thorough process evaluation on both intervention and control schools. Another limitation is the dependence upon self-reported student data. It proved infeasible to also collect parent/teacher data with respect to student health behavioural changes.

Strengths of the study are that within the intervention adolescent health is taken into account in a comprehensive manner, meaning that the focus was not placed merely upon one or two health topics, but on the understanding meaningful health promotion and education should entail the whole of health related behaviours in an integrated fashion. This differs significantly from the way health promotion and health education is commonly integrated into secondary schools in The Netherlands, namely via a disjointed, sporadic and non-evidence-based manner. Furthermore, the monitoring and annual feedback function of the student questionnaire aids to provide the schools with specific, tailored health promoting school policies.

## Acknowledgements and funding

The authors would like to recognize the value the contribution of the pilot school and the four participating schools in this exploratory study. Also, the authors would like to thank several organizations for their value in developing the intervention, namely the Regional Health Services of Central Holland and the different, specialized Dutch knowledge centres Centrum Maliebaan, Stivoro, and the Trimbos Instituut.

This study is co-funded with the help of a regional governmental grant.

## Competing interests

The authors declare that they have no competing interests.

## Ethics approval

This study has been cleared of a need for review by the Institutional Review Board of the University Medical Centre Utrecht, The Netherlands. METC-protocol number 11-397/C.

## Reviewers

**Goof Buijs**, Msc, Manager SHE Network (Schools for Health in Europe) and senior researcher/staff NIGZ (Netherlands Institute for Health Promotion) Woerden, The Netherlands.

**Cécile Rousseau**, Professor, McGill University, CSSS de la Montagne (CLSC Park Extension), Youth Mental Health, Montreal, Quebec, Canada.

**Tetine Lynn Sentell**, PhD, Assistant Professor, Health Policy and Management, 1960 East West Road, Biomed D104G, Honolulu, HI 96822.

## Figures and Tables

**Table 1:** Variables measured in the questionnaire.

Factor	Operational variable
School level	
School	Coded: 1, 2, 3 or 4
Individual/student level	
Socio-demographic characteristics	
Ethnicity*	Dutch, Moroccan, Turkish, Surinamese, Netherlands Antilles, other.
Socio-economic status*	Family affluence scale (FAS) [39]
School level*	Level 1 (‘VMBO’) to level 3 (‘VWO’)
Age*	
Gender*	M/F
Behaviour	
Ever alcohol use*	Yes/No
Recent alcohol use*	Yes/No
Frequency of recent alcohol use*	‘Number of times ever’/‘Number of times per month’
Ever smoked*	Yes/No
Active smoker*	Yes/No
Frequency of smoking*	‘Number of times per week’/‘Quantity per week’
Ever cannabis use*	Yes/No
Recent cannabis use*	Yes/No
Frequency of cannabis use*	‘Number of times ever’/‘Number of times per month’
Breakfast habits*	Number of days per week
Fruit consumption*	Portions per week
Vegetable consumption*	Number of days per week
Physical activity*	At least on average one hour a day moderate to intense physical activity
Recent bullying*	More than once per month
Recent, frequent bullying*	More than once per week
Recent being bullied*	More than once per month
Recent, frequent being bullied*	More than once per week
Ever had intercourse*	Yes/No
Age of first intercourse debut*	Age of student
Contraceptive use behaviour*	Range from ‘Always’ to ‘Never’ (5 point Likert scale)
Ever had STD*	Yes/No/Unsure
Time spent watching television*	Hours per week
Time spent on pc/internet*	Hours per week
Compulsive pc/internet use	Compulsive internet use scale (CIUS) [40]
Time spent (online) gaming*	Hours per week
Compulsive (online) gaming	Videogame addition test (VAT) [41]
Health outcome	
Physical health*	Body mass index (BMI)
Psychosocial health	Strengths and difficulties questionnaire (SDQ)* [42, 43]
	Self efficacy measure [44]

*Variables directly based on the Dutch HBSC questionnaire.
